# Screening for bovine leukocyte adhesion deficiency, deficiency of uridine monophosphate synthase, complex vertebral malformation, bovine citrullinaemia, and factor XI deficiency in Holstein cows reared in Turkey

**DOI:** 10.1186/1751-0147-52-56

**Published:** 2010-10-07

**Authors:** Hasan Meydan, Mehmet A Yildiz, Jørgen S Agerholm

**Affiliations:** 1 Animal Sciences, Faculty of Agriculture, Ankara University, 06110, Dışkapı, Ankara, Turkey; 2 Department of Large Animal Sciences, Faculty of Life Sciences, University of Copenhagen, Dyrlaegevej 68, DK-1870 Frederiksberg C, Denmark

## Abstract

**Background:**

Bovine leukocyte adhesion deficiency (BLAD), deficiency of uridine monophosphate synthase (DUMPS), complex vertebral malformation (CVM), bovine citrullinaemia (BC) and factor XI deficiency (FXID) are autosomal recessive hereditary disorders, which have had significant economic impact on dairy cattle breeding worldwide. In this study, 350 Holstein cows reared in Turkey were screened for BLAD, DUMPS, CVM, BC and FXID genotypes to obtain an indication on the importance of these defects in Turkish Holsteins.

**Methods:**

Genomic DNA was obtained from blood and the amplicons of BLAD, DUMPS, CVM, BC and FXID were obtained by using PCR. PCR products were digested with *Taq*I, *Ava*I and *Ava*II restriction enzymes for BLAD, DUMPS, and BC, respectively. These digested products and PCR product of FXID were analyzed by agarose gel electrophoresis stained with ethidium bromide. CVM genotypes were detected by DNA sequencing. Additionally, all genotypes were confirmed by DNA sequencing to determine whether there was a mutant allele or not.

**Results:**

Fourteen BLAD, twelve CVM and four FXID carriers were found among the 350 Holstein cows examined, while carriers of DUMPS and BC were not detected. The mutant allele frequencies were calculated as 0.02, 0.017, and 0.006 for BLAD, CVM and FXID, respectively with corresponding carrier prevalence of 4.0% (BLAD), 3.4% (CVM) and 1.2% (FXID).

**Conclusion:**

This study demonstrates that carriers of BLAD, CVM and FXID are present in the Turkish Holstein population, although at a low frequency. The actual number of clinical cases is unknown, but sporadic cases may appear. As artificial insemination is widely used in dairy cattle breeding, carriers of BLAD, CVM and FXID are likely present within the population of breeding sires. It is recommended to screen breeding sires for these defective genes in order to avoid an unwanted spread within the population.

## Background

Modern breeding of dairy cattle increasingly involves programs based on international trade of semen from elite bulls with high genetic merit. With the widespread use of advanced reproductive technologies, including artificial insemination and multiple ovulation embryo transfer, individual bulls are able to quickly sire thousands of calves in many countries [1].

In animal breeding, genetic disorders are one of the most important issues for breeders. Due to the negative influence of such disorders on animals e.g. through abnormal anatomy or reduced production, breeders and breeding associations need to control the impact on the population.

Known inherited disorders in cattle are mostly caused by autosomal recessively inherited genes. The characteristic feature of autosomal recessive genes is that they are only expressed as a diseased phenotype if both alleles are present. Therefore, unrecognized dissemination of such defective genes is possible and autosomal recessively inherited disorders are of greater concern in cattle breeding than are disorders with dominant inheritance or recessive X-linked inheritance as these are easily recognized [2].

Heterozygous individuals can be identified by different methods such as examination of progeny e.g. by clinical examination or necropsy, analysis of enzyme activity in blood, and genotyping of animals by genomic analysis. Recent developments within molecular genetics have made possible efficient and rapid identification of heterozygous animals by genomic analysis. Knowing the molecular basis of a defect, the direct detection of carriers is possible at the genetic level, thus preventing unintended breeding of the animal [2]. At present, there are identification records for several inherited bovine disorders as e.g. bovine leukocyte adhesion deficiency (BLAD), deficiency of uridine monophosphate synthase (DUMPS), complex vertebral malformation (CVM), bovine citrullinaemia (BC), and factor XI deficiency (FXID) [1,2].

BLAD is a lethal autosomal recessive disorder and known to affect Holstein cattle breed throughout the world. BLAD is characterized by greatly reduced expression of the heterodimeric *β_2 _*integrin adhesion molecules on leukocytes resulting in multiple defects in leukocyte function. Defective leukocyte adherence leads to inadequate mucosal immunity. BLAD affected cattle have severe and recurrent mucosal infections such as pneumonia, gingivitis, periodontitis, loss of teeth, papillomatosis, dermatophytosis, impaired pus formation, delayed wound healing, and stunted growth [3,4]. Most cattle with BLAD die without having the diagnosis established, probably before one year of age. Some cows survive for more than two years. However their reproduction and milk performances are poor. Consequently, BLAD is an economically important disease emphasizing the need for genetic screening to eliminate the mutant allele from the population. The molecular basis of BLAD is a single point mutation (A→G) of nucleotide 383 in the CD18 gene located bovine chromosome 1 [2,5-8].

DUMPS is a hereditary lethal autosomal recessive disorder in Holstein cattle causing early embryonic mortality during implantation in the uterus. DUMPS interfere with pyrimidine biosynthesis and is inherited as a single autosomal locus with two-alleles [9-11]. In mammalian cells, the last step of pyrimidine nucleotide synthesis involves the conversion of orotate to uridine monophosphate synthase (UMP) and is catalyzed by UMP synthase enzyme. UMP synthase is necessary for the *de novo *synthesis of pyrimidine nucleotides, which are constituents of DNA and RNA. Growth and development of the homozygous recessive is arrested leading to embryonic mortality around 40 days post-conception. DUMPS is caused by single point mutation (C→T) at codon 405 within exon 5. The UMP synthase gene was mapped to the bovine chromosome 1 [12-14].

CVM is a recessively inherited disorder with onset during embryonic development leading to frequent abortion of affected fetuses or perinatal death associated with vertebral anomalies. The syndrome was first discovered in the Danish Holstein population [15,16]. Typical signs of CVM are a shortened neck and bilateral, symmetrical, moderate contraction of the carpal joints, severe contraction and slight lateral rotation of the fetlock joints. The hind limbs show marked bilateral, symmetrical contraction of the fetlocks with medial rotation of distal limbs. Malformation of multiple vertebrae, mainly involving those at the cervico-thoracic junction, is a common feature [15,17-19]. The US Holstein-Friesian sire Penstate Ivanhoe Star (US1441440) has been identified as the common ancestor bull and his son Carlin-M Ivanhoe Bell (US1667366) that has been used in dairy cattle breeding worldwide for two decades due to the superior lactation performance of his daughters [1,2,16]. CVM is caused by a missense mutation in the gene *SLC35A3 *(solute carrier family 35 member 3) coding an uridinediphosphate-N-acetylglucosamine transporter. A single base transversion of guanine to thymine has been located in the abnormal allele at position 559 in the gene *SLC35A3 *located bovine chromosome 3 [20,21].

BC in Holsteins is an autosomal recessively inherited disease that was first described in the Australian Holstein population [22-24]. This genetic disorder prevents the synthesis of argininosuccinate synthetase, the enzyme that catalyses the conversion of citrulline and aspartate to argininosuccinate at the consumption of ATP. Cattle affected by BC appear normal immediately after birth. However, by the 2nd day of life they become depressed and feed poorly. By the 3rd day, they are often seen aimlessly wandering about their enclosure or standing with their head pressed against a fence or wall. Between the 3rd and 5th day, the disease progresses rapidly. The calves appear to be blind and finally collapse. Homozygous cattle die during the first 7 days of life. BC is caused by a transition of cytosine (CGA/arginine) into thymine (TGA/STOP codon) at codon 86 of the gene coding for argininosuccinate synthase leading to impaired urea cycle. The BC gene was mapped to the bovine chromosome 11 [25,26]. BC was disseminated throughout the Australian Holstein population following importation of semen from the US sire Linmack Kriss King [27,28].

Factor XI is one of more than a dozen proteins involved in blood clotting. FXID has been identified in several species of mammals, including humans, dogs and cattle [29-32]. FXID may result in prolonged bleeding from the umbilical cord and anemia. Prolonged oozing of blood following dehorning or castration may also be observed. Affected cows frequently have pink-colored colostrum. Blood in the milk led to the identification of this condition in a British dairy herd [33]. Additionally, FXID causes reduced reproduction performance and the affected animals appear to be more susceptible to diseases such as pneumonia, mastitis and metritis. Therefore, the presence of this genetic defect may have a significant economic impact on the dairy industry [34,35]. Affected animals may survive for years with no overt clinical signs, even though they appear to have a higher mortality and morbidity rate. The causative mutation for FXID have been identified by the authors [32] who found that the mutation consists of a 76 bp segment insertion into exon 12 in bovine chromosome 27.

This paper provides an overview of BLAD, DUMPS, CVM, BC and FXID in Holstein cows reared in Turkey. The goal of this study is to estimate the prevalence of BLAD, DUMPS, CVM, BC and FXID in Turkish Holstein cattle using DNA based tests.

## Materials and methods

Three hundred and fifty Holstein cows from the provinces of Ankara (n = 225) located in center of Anatolia and Şanlıurfa (n = 125) located in South East Anatolia in Turkey were sampled at random. Random sampling was done on the Holstein cows that were brought to Ankara and Şanlıurfa slaughterhouses between the years 2007 and 2009 to be slaughtered.

Blood samples were collected from the jugular vein into EDTA containing tubes, transported to the laboratory and stored at -20°C until genomic DNA extraction, which was carried out using a salting-out method [36]. Genomic DNA was stored at 4°C until analysis.

Genotyping for BLAD, DUMPS and BC was done using PCR-RFLP methods. CVM genotypes were identified by DNA sequencing. The genotypes of FXID were detected by PCR methods. The primers, PCR profiles, PCR product sizes and restriction enzymes used for identification of each genetic disorder are shown in Table 1.

**Table 1:** Primers, PCR profiles, PCR product sizes and restriction enzymes (RE) used for identification of bovine leukocyte adhesion deficiency (BLAD), deficiency of uridine monophosphate synthase (DUMPS), complex vertebral malformation (CVM), bovine citrullinaemia (BC) and factor XI deficiency (FIXD)

Genetic disorder	Primer	PCR profile			PCR product size	RE
**BLAD** Newly designed	F: 5' GAATAGGCATCCTGCATCATATCCACCA 3' R: 5' CTTGGGGTTTCAGGGGAAGATGGAGTAG 3'	94°C	03 m	33 cycle	357 bp	*Taq*I
		94°C	30 s			
		65°C	30 s			
		72°C	30 s			
		72°C	05 m			

**DUMPS** [13]	F: 5' GCAAATGGCTGAAGAACATTCTG 3' R: 5' GCTTCTAACTGAACTCCTCGAGT 3'	94°C	05 m	40 cycle	108 bp	*Ava*I
		94°C	60 s			
		58°C	60 s			
		72°C	90 s			
		72°C	05 m			

**CVM** Newly designed	F: 5' CAGATTCTCAAGAGCTTAATTCTA 3' R: 5' TATTTGCAACAACAAGCAGTT 3'	94°C	05 m	35 cycle	281 bp	-
		94°C	45 s			
		52°C	45 s			
		72°C	60 s			
		72°C	10 m			

**BC** [25]	F: 5' GGCCAGGGACCGTGTTCATTGAGGACATC 3' R: 5' TTCCTGGGACCCCGTGAGACACATACTTG 3'	94°C	03 m	35 cycle	198 bp	*Ava*II
		94°C	30 s			
		57°C	30 s			
		72°C	30 s			
		72°C	10 m			

**FXID** [32]	F: 5' CCCACTGGCTAGGAATCGTT 3' R: 5' CAAGGCAATGTCATATCCAC 3'	95°C	10 m	34 cycle	320 bp	-
		95°C	30 s			
		55°C	60 s			
		72°C	30 s			
		72°C	10 m			

BLAD, BC and FXID genotypes were determined using 2% agarose gel electrophoresis stained with ethidium bromide. DUMPS genotypes were visualized on 4% MetaPhor agarose gel electrophoresis stained with ethidium bromide due to the limited size of the digested fragments. For CVM screening, the samples were sequenced by a Big Dye Terminator chemistry on an ABI 3100 Avant Automated DNA Sequencer (Applied Biosystems, Foster City, CA, USA). The DNA sequences were analyzed using the Sequencing Analysis Software Version 3.3 (Applied Biosystems). DNA of known carriers of BLAD and DUMPS as a control were obtained from Dr. A Wohlke, Institute for Animal Breeding and Genetics, University of Veterinary Medicine Hannover, Hannover, Germany. CVM carriers and affected DNA samples as a control were supplied by Dr. J S Agerholm (also co-author in this paper) and DNA samples of FXID carriers and affected animals as a control were provided by Dr. J E Beever, Department of Animal Sciences, University of Illinois-Urbana, USA.

The all genotypes were confirmed by DNA sequencing. After gel electrophoresis, the amplicons were purified using a QIAamp Mini Kit (QIAGEN, Valencia, CA, USA) and sequenced by a Big Dye Terminator chemistry on an ABI 3100 Avant Automated DNA Sequencer. Sequencing was done by Refgen Biotechnology (http://www.refgen.com).

The mutant gene frequency of the BLAD, CVM and FXID was estimated by counting the number of genes [37].

## Results

**Figure 1:**
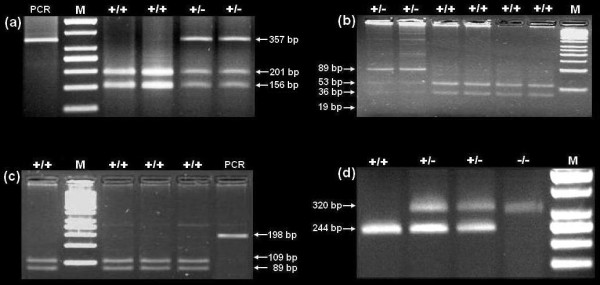
**Illustration of BLAD, DUMPS, BC and FXID genotypes on agarose gels**. (a) genotypes of bovine leukocyte adhesion deficiency (BLAD), (b) genotypes of deficiency of uridine monophosphate synthase (DUMPS), (c) genotypes of bovine citrullinaemia (BC) and (d) genotypes of factor XI deficiency (FXID). +/+: unaffected genotype, +/-: carrier genotype, -/-: affected genotype, M: DNA Ladder.

The primers listed in Table 1 successfully amplified the DNA fragments of 357 bp, 108 bp, 281 bp, 198 bp and 320 bp for BLAD, DUMPS, CVM, BC and FXID, respectively. The PCR products of BLAD, DUMPS and BC were digested with *Taq*I, *Ava*I and *Ava*II restriction enzymes, respectively. After digestion of the PCR products, the normal BLAD allele in unaffected cattle produced two fragments of 156 bp and 201 bp. BLAD carriers exhibit three fragments of 156 bp, 201 bp and 357 bp. In unaffected animals, normal DUMPS allele exhibits three fragments of 53 bp, 36 bp and 19 bp. DUMPS carriers gave four fragments of 89 bp, 53 bp, 36 bp and 19 bp. The normal allele of BC produced two fragments of 109 bp and 89 bp. After the PCR, the normal FXID allele in unaffected animals produced a single 244 bp fragment. In homozygous affected animals, the fragment had a length of 320 bp and FXID carriers exhibited two fragments of 244 bp and 320 bp (Fig. 1.).

Among the 350 Holstein cows reared in Turkey, 14 BLAD, 12 CVM and 4 FXID carriers were found, while carriers of DUMPS and BC were not detected. The mutant allele frequency and the prevalence of BLAD carriers were 0.02% and 4.0%, respectively. The mutant allele frequency and the prevalence of CVM carriers were 0.017% and 3.4%, respectively. The frequency of the mutant FXID allele and the prevalence of carriers were calculated as 0.006% and 1.2%, respectively.

**Figure 2:**
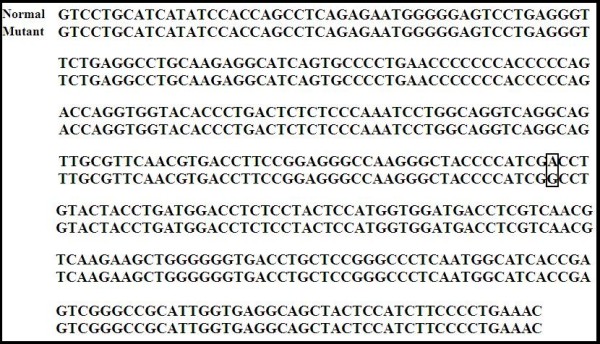
**Alignment of bovine leukocyte adhesion deficiency (BLAD) sequences from normal and mutant (FJ853493) BLAD alleles**. The mutation consists of a single point mutation of nucleotide 383 in the CD18 gene. The box indicates the single point mutation site (A→G).

**Figure 3:**
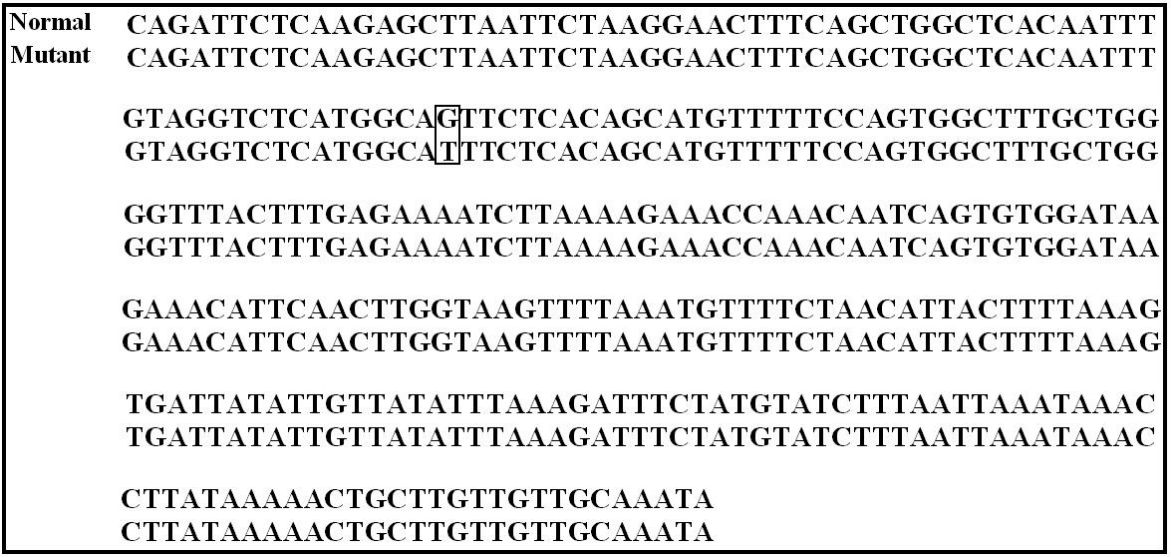
**Alignment of complex vertebral malformation (CVM) sequences from normal (HM183012) and mutant (HM183013) CVM alleles**. The mutation consists of a single point mutation of nucleotide 559 in the SLC35A3 gene. The box indicates the single point mutation site (G→T).

**Figure 4:**
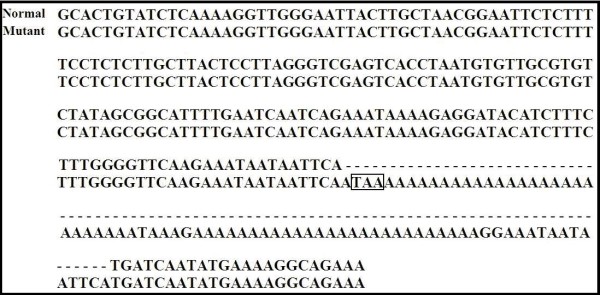
**Alignment of bovine factor XI deficiency (FXID) sequences from normal (FJ853492) and mutant (GQ144406) FXID alleles**. The mutation consists of a 76 bp segment insertion into exon 12 of FXI gene. The insertion consists of long strings of adenine (A) bases and contains a STOP codon (TAA). The box indicates the premature STOP codon generated as a result of the insertion.

All genotypes were confirmed by doing partial sequencing. The nucleotide sequences were deposited in GenBank with accession numbers FJ853493 for BLAD, HM183012 and HM183013 for CVM, FJ853494 for BC, FJ853492 and GQ144406 for FXID. The result of sequencing for the mutant BLAD allele was confirmed a single point mutation at the nucleotide 383 in the CD18 gene as reported before [38] (Fig. 2.). The sequencing of the mutant CVM allele was consistent with the previous report [20] (Fig. 3.). The mutant FXID allele sequencing result was also consistent with a prior report [32] describing a mutation consisting of a 76 bp insertion containing poly adenine sequences along with a STOP codon (TAA) (Fig. 4.).

## Discussion

Identification of the molecular basis for genetic disorders enables a rapid screening of breeding populations in order to eliminate the carriers from the population of breeding sires, thus decreasing the number of affected progeny. This study shows that PCR-RFLP analysis is a strong and reliable method for identification of BLAD, DUMPS, BC and FXID.

Previously, carriers of BLAD have been identified in Turkish Holstein cattle [39,40]. The frequency of mutant BLAD allele was estimated to 0.084 [39] and 0.035 [40]. The frequency determined in the present study is almost three times higher than that found by Akyüz and Ertuğrul [39] while it is almost similar to that reported by Meydan, Yildiz and Özdil [40]. The reason for this discrepancy is probably sampling of of different populations i.e. cattle in different regions, although it may also be influences by the use of carrier sires over time. The prevalence of carriers found in this study (4.0%) was similar to that found by in Brazil (2.8%) [7], Japan (4.0%) [41], USA (4.0%) [42,43], Poland (3.0%) [44] and Iran (3.3%) [45].

In Turkey, no carriers were found for DUMPS or BC similar to previous studies [40,46,47]. The results obtained in this study also correspond to findings in Poland [11], Czech Republic [14], Germany [25], India [26], Iran [48], and Romania [49]. By contrast, the frequency of the mutant allele for DUMPS has been estimated at 1-2% in US Holstein cattle [50], 0.96% in Argentinian Holstein bulls and 0.11% in Argentinian Holstein cows [51] in studies performed during the 1990s. Similarly, carriers of BC have been detected in USA and Australia [17,20]. Discrepancies between studies probably reflect differences in the use of affected breeding lines between different regions.

CVM in cattle has been reported in countries such as Czech Republic [14], Denmark [15], Poland [21], USA [52], United Kingdom [53], Japan [54], Iran [55], and Sweden [56]. However, no case of CVM was previously reported in Holstein cattle reared in Turkey. As a first report for CVM, this study found that the mutant CVM allele frequency in Holstein cows reared in Turkey is 0.017% and the prevalence of carrier cattle is 3.4%. The prevalence found in this study is very low compared to Denmark (31.0%) [20], Poland (24.8%) [21], Japan (32.5%) [54], Sweden (23.0%) [56], and Germany (13.2%) [57]. Since the early 2000s, most countries have developed breeding programs for CVM to decrease the prevalence of carriers. However, in a recent report [21] 150 CVM carriers (24.8%) were identified from 605 Polish Holstein sires. In another recent study performed in 200 Japanese Holstein cows, 26 animals were CVM carriers (13.0%) [58]. Hence, in some Holstein populations, the frequency of CVM carriers still seems to be high.

In previous studies on FXID in Turkey, the prevalence of the carriers was calculated as 1.8% [59] and 1.2% [47] based on the examination of 225 and 170 Holstein cattle, respectively. The prevalence of carrier cattle (1.2%) in the present study was quite similar compared to those studies. The similarity of FXID prevalence within the three Turkish studies can be explained that the probability of carrying the mutant FXID allele of bulls used for artificial insemination is the almost same for these studies. The prevalence in this study is also similar to that observed in other reports [32,34,35].

In this study, more cattle were tested for BLAD, DUMPS and FXID than previous studies [39,40,46,47,59] and sampled in different populations (Ankara and Şanlıurfa) in Turkey. Also, in this study all genotypes were confirmed by DNA sequencing in order to make sure that there was a mutant allele. Moreover, we wanted to make sure that there were no DUMPS and BC carriers in Turkey although carrier of DUMPS and BC was not found in the previous studies [40,46,47] in Turkey.

The actual number of clinical cases of BLAD, CVM and FXID in Turkey is unknown, but as carriers were found, sporadic cases probably appear. As artificial insemination is widely used in dairy cattle breeding, carriers of BLAD, CVM and FXID are likely present within the population of breeding sires. It is recommended to screen breeding sires for these defects to avoid an unrecognized spread of the defective genes within the population.

## Conclusions

The study demonstrates that carriers of BLAD, CVM and FXID are present in the Turkish Holstein population, although at a low frequency. By contrast, carriers of BC and DUMPS were not detected. This is the first report on CVM in Holstein cattle reared in Turkey. PCR-RFLP analysis was used for genomic analyses and was found to be a strong and reliable method for identification of BLAD, DUMPS, BC and FXID in Holstein cattle. The study demonstrates a need for further examination of more cattle in Turkey, preferably by testing the breeding sires to avoid unrecognized spread of genetic disorders.

## Competing interests

The authors declare that they have no competing interests.

## Authors' contributions

HM collected the blood samples, carried out the extraction of genomic DNA, PCR and DNA sequencing and participated in the writing of the manuscript. MAY conceived of the study, participated in its design and coordination, performed the statistical analysis and participated in the writing of the manuscript. JSA participated in writing of the manuscript and interpretation of results. All authors read and approved the final manuscript.
